# T159. ASSOCIATION BETWEEN VITAMIN D INSUFFICIENCY AND METABOLIC SYNDROME IN PATIENTS WITH PSYCHOTIC DISORDERS

**DOI:** 10.1093/schbul/sby016.435

**Published:** 2018-04-01

**Authors:** Sung-Wan Kim, Taeyoung Yoo, Jung Jin Kim, Jae-Kyeong Kim, Ji-Eun Hong, Ju-Yeon Lee, Jae-Min Kim, G Paul Amminger, Michael Berk, Jin-Sang Yoon

**Affiliations:** 1 Chonnam National University Medical School; 2 The Catholic University of Korea Kangnam, St. Mary’s Hospital; 3 Mindlink, Gwangju Bukgu Mental Health Center; 4 Gwangju Mental Health Commission; 5 Chonnam National University Medical School, Gwangju Bukgu Community Mental Health Center; 6 Orygen - The National Centre of Excellence in Youth Mental Health; 7 Deakin University, School of Medicine

## Abstract

**Background:**

Vitamin D levels are low in patients with schizophrenia. This study examined the association between vitamin D and metabolic syndrome in patients with psychotic disorders.

**Methods:**

The study enrolled 302 community-dwelling patients with psychotic disorders. Sociodemographic and clinical characteristics, including blood pressure, physical activity, and dietary habit were gathered. Laboratory examinations included vitamin D, lipid profile, fasting plasma glucose, HbA1c, liver function, and renal function. Vitamin D insufficiency was defined as < 20 ng/ml. Clinical characteristics associated with vitamin D insufficiency were identified.

**Results:**

Among the 302 participants, 236 patients (78.1%) had a vitamin D insufficiency and 97 (32.1%) had metabolic syndrome. Vitamin D insufficiency was significantly associated with the presence of metabolic syndrome (p = 0.006) and hypertension (p = 0.017). Significant increases in triglycerides and alanine transaminase were observed in the group with a vitamin D insufficiency (p = 0.002 and 0.011, respectively). After adjusting for physical activity and dietary habit scores, vitamin D insufficiency remained significantly associated with metabolic syndrome and hypertension.

**Discussion:**

Vitamin D insufficiency was associated with metabolic syndrome and was particularly associated with high blood pressure, although the nature, direction and implications of this association are unclear.

